# Supplementary material for: Repeated evidence that the accelerated evolution of sperm is associated with their fertilization function
Source: Proc Biol Sci. 2020 Aug 5;287(1932):20201286. doi: 10.1098/rspb.2020.1286 (PMC7575512; doi:10.1098/rspb.2020.1286)

**Electronic Supplementary Material**

**Repeated evidence that the accelerated evolution of sperm is associated with their fertilization function**

John L. Fitzpatrick^1,2*^, C. Daisy Bridge^2^, Rhonda R. Snook^1^

^1^ Department of Zoology, Stockholm University, Svante Arrhenius väg 18B, SE-10691, Sweden

^2^ Faculty of Life Sciences, Michael Smith Building, University of Manchester, M13 9PT, UK

**Supplementary Methods**

**Phylogenetic analyses**

Prior to evolutionary rates analyse we assessed whether traits of interest conform to a Brownian pattern of evolution, a key assumption of the rates analyses used in our study (1). We statistically compared alternative models of evolution to determine if Brownian motion models best explained the evolution of each trait. A subset of traits that conformed to a Brownian motion model of evolution were then used to compare rates of evolutionary divergence in reproductive and somatic traits for both taxonomic groups. All phylogenetic analyses were performed using the packages specified below in RStudio v3.5.1 statistical software (R Development Core Team, 2018). All data were converted to the same scale (μm) and log10-transformed prior to phylogenetic analyses to create unit-less variables and allow for the evolutionary change of different traits to be compared on a common scale (1).

***Comparing evolutionary models***

We contrasted directly if trait evolution best fit a Brownian motion (BM), Ornstein-Uhlenbeck (OU), or Early-burst (EB) model of evolution using the *fitContinuous* function in the R package *geiger* (2). For *Drosophila* from the *obscura* group BM was either the best fit model or statistically indistinguishable from alternative models (Δ_i_ <4.0) for all of the traits examined, except for female wing length (Table S2a). For Lepidoptera comparison of alternative evolutionary models revealed that the BM model was the best fit (lowest AICc) and highest weighted model for all traits examined (ESM Table S2b), although both OU and EB models had comparable AICc values (Δ_i_ <4.0, 3).

***Comparing evolutionary rates***

We focused our analyses only on traits that exhibited phylogenetic signal consistent with a Brownian motion (BM) process based on the comparison of alternative evolutionary models (see Tables S2). Significant differences between the observed and common models reveal that traits are evolving at different evolutionary rates, but not how rates of evolution differ among traits (in cases where more than two traits are examined). Therefore, in analyses that evaluated three or more traits simultaneously, we used pairwise comparisons of all trait combinations to identify which traits were evolving at different evolutionary rates (1). The results of these pairwise comparisons are presented in the main text in Figure 1 and the statistical output is presented in Table S3.

When comparing evolutionary rates, the Nelder-Mead optimization function was used for most models, which facilitated model convergence. We assumed evolutionary covariation in the observed evolutionary rate matrices among traits in all models, which is typical among phenotypic traits (1, 4). Since we lacked individual-specific data and data on measurement error for all of the traits assessed, we did not incorporate intraspecific trait covariance or measurement error into our analyses.

***Supplemental Lepidoptera Analyses***

For Lepidoptera the analyses presented in the main text were constrained by the mismatch between species where data was available and species that were present in Kawahara et al.’s (5) phylogeny. This mismatch meant that we were able to compare rates of evolution between different sperm types and forewing length for only 12 of the 135 species in our original dataset. Therefore, we constructed an additional phylogeny to examine a wider number of Lepidoptera species (see below). In so doing, we were also able to contrast rates of evolution between sperm types among 135 Lepidopteran species.

We used the mitochondrial gene cytochrome c oxidase subunit I (COI) to build a Lepidoptera phylogeny. Fossil evidence suggests that Lepidoptera arose ~190 Mya (6). The relatively ancient timescale of the Lepidoptera origin makes it challenging to determine species relationships when using mitochondrial genes with high substitution rate (like COI). However, the mitochondrial gene COI was the only available sequence for roughly three quarters of the species in our dataset. Overall, COI was available for 138 Lepidoptera species (50 butterflies and 88 moths) where we also had data on sperm lengths. Among these species, COI was the only available gene for the moth species in our dataset, and only one additional gene (16S ribosomal RNA gene) was available for a subset (n = 34) of butterfly species in our dataset. Therefore, despite the caveats with interpreting results from a COI-derived phylogeny for Lepidoptera, we performed an additional set of analyses to determine if the pattern we observed in the 12 species that were present in Kawahara et al.’s (5) phylogeny (presented in the main text) are consistent with the pattern detected across a broader taxonomic scale. However, these constraints meant that we did not interpret the absolute values for the rate of evolution for each trait, as these will be dependent on the phylogeny used in the analysis. Instead we focused on the relative patterns of evolutionary rates across the traits, which are unlikely to change qualitatively as the traits are compared on the same overall phylogenetic topology in all analyses. Data and accession numbers are presented in ESM Table S1.

Nucleotides sequences were aligned in Mesquite version 3.40 (7) using the MUSCLE extension for this program (8). Aligned gene sequences were then visually inspected in Mesquite to identify any problematic alignments. Sequences for three butterfly species (*Heliconius erato*, *H. melpomene*, and *Tithorea harmonia*) did not align with, and caused misalignment of, the remaining species. Therefore, these three species were excluded from the phylogeny and subsequent analyses, reducing the Lepidoptera dataset to 135 species (47 butterflies and 88 moths). Following alignment, we used jModelTest v2.1.6 (9) to identify the best-fit nucleotide substitution model for each sequence. The Akaike Information Criterion corrected for sample size (AICc) was used to distinguish between three substitution models. The best-fit nucleotide substitution models was GTR+Γ+I. However, phylogenetic construction using jModelTest models failed to converge across a range of parameter settings. Therefore, we simplified the substitution models to GTR, which led to convergence of the Bayesian chain.

The Lepidoptera phylogeny was constructed using the Bayesian tree-building analysis software BEAUTi v1.8.2 and BEAST v1.8.2 (10) using a relaxed uncorrelated lognormal clock, default BEAST values for sequence-evolution parameters, a Yule speciation process, and no priors set for root dates. Priors were set to a uniform distribution with an initial value of 0.6 and upper and lower values of 1 and 0, respectively. The MCMC (Markov Chain Monte Carlo) simulation chain generated by BEAST was set to a length of 30 million steps with parameters logged every 30,000 steps. Stationarity was verified using Tracer v1.5 software (11), based on inspection of the posterior distribution of the traces and an effective sample size (ESS) exceeding 200 for each parameter. A maximum clade credibility (MCC) tree was generated using mean node heights and a 20% burn-in and a posterior probability limit of 0.90 using TreeAnnotator v1.8.1 (10) and viewed using FigTree v1.4.2 (10). To verify the resulting phylogeny, we re-ran the steps described above using alternative staring points and a greater number of sequences for butterflies (COI and 16S). The topology remained entirely or largely consistent regardless of how the construction method or input sequences.

Using this COI-derived Lepidoptera phylogeny, we compared rates of evolution between fertile and non-fertile sperm among our now expanded Lepidoptera dataset. Consistent with the pattern reported in the main text, fertile sperm evolved faster than non-fertile sperm: rates of fertile sperm evolution were 2.9 times greater than rates of non-fertile sperm evolution across the 135 species of Lepidoptera we examined (ESM Table S4).

**Supplementary References**

1. Adams DC. 2013 Comparing evolutionary rates for different phenotypic traits on a phylogeny using likelihood. *Syst. Biol.* **62**, 181–192.
2. Harmon LJ, Weir JT, Brock CD, Glor RE, Challenger W. 2008. GEIGER: investigating evolutionary radiations. *Bioinform.* **24**, 129–131.
3. Burnham KP, Anderson DR. 2002 Model selection and multimodel inference: A practical information-theoretic approach. 2nd ed. Springer-Verlag, New York.
4. Simmons LW, Fitzpatrick JL. 2016 Sperm competition and the coevolution of pre- and postcopulatory traits: Weapons evolve faster than testes among onthophagine dung beetles. *Evol*. **70**, 998–1008.
5. Kawahara AY, Plotkin D, Espeland M, Meusemann K, Toussaint EFA, Donath A, Gimnich F, Frandsen PB, Zwick A, dos Reis M, Barber JR, Peters RS, Liu S, Zhou X, Mayer C, Podsiadlowski L, Storer C, Yack JE, Misof B, Breinholt JW. 2019 Phylogenomics reveals the evolutionary timing and pattern of butterflies and moths. *Proc. Nat. Acad. Sci. USA* **116**, 22657-22663. doi: 10.1073/pnas.1907847116
6. Mitter C, Davis DR, Cummings MP. 2017 Phylogeny and evolution of Lepidoptera. *Annu. Rev. Entomol*. **62**, 265–283.
7. Maddison WP, Maddison DR. 2018 Mesquite: a modular system for evolutionary analysis. Version 3.40 <http://mesquiteproject.org>.
8. Edgar RC. 2004 MUSCLE: multiple sequence alignment with high accuracy and high throughput. *Nucleic Acid Res.* **32**, 1792–1797.
9. Darriba D, Taboada GL, Doallo R, Posada D. 2012. jModelTest 2: more models, new heuristics and parallel computing. *Nat. Methods* **9**, 772.
10. Drummond AJ, Suchard MA, Xie D, Rambaut A. 2012 Baysesian phylogenetics with BEAUti and the BEAST 1.7. *Mol. Biol. Evol.* **29**, 1969–1973.
11. Rambaut A, Drummond AJ, Xie D, Baele G, Suchard MA. 2018 Posterior Summarization in Bayesian Phylogenetics Using Tracer 1.7. *Syst Biol*. **67**, 901‐904. doi:10.1093/sysbio/syy032

**ESM Table S1**: Species trait data, accession numbers, and phylogenies for the *Drosophila* species from the *obscura* group and Lepidoptera examined in this study (see Excel document).

**ESM Table S2.** Comparison of model parameters and fit for sperm length, female reproductive organ length and somatic traits examined under Brownian motion (BM), Ornstein-Uhlenbeck (OU) and Early-burst (EB) evolutionary models for (a) *Drosophila* from the *obscura* group and (b) Lepidoptera. Fertile (eusperm or eupyrene) and non-fertile (parasperm or apyrene) head length (L_head_), flagellum length (L_flagellum_), and total length (L_total_) were assessed. The Brownian rate parameter, σ^2^, selection strength parameter, α, and rate of evolutionary change parameter, *a*, are presented for the BM, OU and EB models, respectively. The maximum likelihood estimates (lnL) and sample size corrected Akaike Information Criterion (AICc) values are presented. Models were compared using the AICc values presented in the table. To compares model fits, for each trait we report the value of delta AICc, Δ_i_, and the Akaike weights, ω_i_, which indicate the strength of evidence for each model. Δ_i_ < 4 indicates statically equivalent models of evolution (3).

|  |  | **Brownian Motion Model** | | | | |  | **Ornstein-Uhlenbeck Model** | | | | |  | **Early Burst Model** | | | | |
| --- | --- | --- | --- | --- | --- | --- | --- | --- | --- | --- | --- | --- | --- | --- | --- | --- | --- | --- |
| **Trait** | | **σ^2^** | **lnL** | **AICc** | **Δ_i_** | **ω_i_** |  | **α** | **lnL** | **AICc** | **Δ_i_** | **ω_i_** |  | ***a*** | **lnL** | **AICc** | **Δ_i_** | **ω_i_** |
| **(a) *Drosophila* from the *obscura* group** | | | | |  |  |  |  |  |  |  |  |  |  |  |  |  |  |
|  | Fertile sperm L_head_ | 2.15 | -4.63 | 14.59 | 1.84 | 0.17 |  | 24.36 | -1.87 | 12.75 | 0.0 | 0.42 |  | 48.73 | -1.87 | 12.75 | <0.001 | 0.42 |
|  | Fertile sperm L_flagellum_ | 1.10 | -0.61 | 6.56 | 0.0 | 0.42 |  | 15.20 | 0.84 | 7.32 | 0.76 | 0.29 |  | 30.41 | 0.84 | 7.32 | 0.76 | 0.29 |
|  | Fertile sperm L_total_ | 0.95 | -0.96 | 6.67 | 2.21 | 0.14 |  | 14.97 | 1.57 | 4.45 | 0.0 | 0.43 |  | 29.9 | 1.57 | 4.45 | <0.001 | 0.43 |
|  | Non-fertile sperm L_head_ | 0.10 | 13.67 | -22.01 | 0.0 | 0.68 |  | 6.40 | 14.06 | -19.12 | 2.89 | 0.16 |  | 12.80 | 14.06 | -19.12 | 2.89 | 0.16 |
|  | Non-fertile sperm L_flagellum_ | 0.43 | 5.00 | -4.66 | 0.0 | 0.59 |  | 13.47 | 5.76 | -2.52 | 2.14 | 0.20 |  | 26.93 | 5.76 | -2.52 | 2.14 | 0.20 |
|  | Non-fertile sperm L_total_ | 0.31 | 9.66 | -14.57 | 0.92 | 0.24 |  | 15.06 | 11.55 | -15.50 | 0.0 | 0.38 |  | 30.11 | 11.55 | -15.50 | <0.001 | 0.38 |
|  | Female thorax length | 0.01 | 38.46 | -72.13 | 0.0 | 0.37 |  | 11.78 | 39.78 | -71.84 | 0.29 | 0.32 |  | *23.56* | 39.78 | -71.84 | 0.29 | 0.32 |
|  | Male thorax length | 0.01 | 36.95 | -69.10 | 0.0 | 0.55 |  | 6.55 | 37.49 | -67.27 | 1.83 | 0.22 |  | 13.10 | 37.49 | -67.27 | 1.83 | 0.22 |
|  | Female wing length | 0.02 | 31.94 | -59.08 | 5.06 | 0.04 |  | 35.68 | 35.93 | -64.14 | 0.0 | 0.48 |  | 71.36 | 35.93 | -64.14 | <0.001 | 0.48 |
|  | Male wing length | 0.03 | 31.12 | -57.44 | 1.79 | 0.17 |  | 17.19 | 33.47 | -59.23 | 0.0 | 0.42 |  | 34.39 | 33.47 | -59.23 | <0.001 | 0.42 |
|  | Seminal receptacle length | 1.09 | -2.43 | 9.86 | 1.03 | 0.23 |  | 15.26 | -0.32 | 8.82 | <0.01 | 0.38 |  | 30.53 | -0.32 | 8.83 | 0.0 | 0.39 |
|  | Spermathecal duct length | 0.89 | -0.53 | 6.26 | 0.38 | <0.01 |  | 2085.4 | 18.12 | -27.58 | 0.0 | 0.56 |  | 1096.6 | 17.90 | -27.13 | 0.46 | 0.44 |
|  |  |  |  |  |  |  |  |  |  |  |  |  |  |  |  |  |  |  |
| **(b) Lepidoptera** | |  |  |  |  |  |  |  |  |  |  |  |  |  |  |  |  |  |
|  | Eupyrene length | 0.05 | 1.81 | 1.70 | 0.0 | 0.69 |  | 2.06 | 2.15 | 4.71 | 3.00 | 0.15 |  | 4.13 | 2.15 | 4.71 | 3.00 | 0.15 |
|  | Apyrene length | 0.14 | 9.89 | -14.44 | 0.0 | 0.76 |  | <0.001 | 9.89 | -10.77 | 3.67 | 0.12 |  | <0.001 | 9.89 | -10.77 | 3.67 | 0.12 |
|  | Forewing length | 0.03 | 4.74 | -4.16 | 0.0 | 0.74 |  | 0.79 | 4.84 | -0.69 | 3.47 | 0.13 |  | 1.58 | 4.84 | -0.69 | 3.47 | 0.13 |

**ESM Table S3.** Pairwise comparisons of evolutionary rates assuming trait covariance in the observed rate matrices for the full models presented in Table 1 of the main text for (a) *Drosophila* from the *obscura* group and (b) Lepidoptera. For each pairwise comparison of fertile (eusperm or eupyrene) and non-fertile (parasperm or apyrene) sperm total length (L_total_), wing and forewing length, and female reproductive organ length (seminal receptacle length for *Drosophila* species), the Log Likelihood values for the observed (LogL_obs_) and common models (LogL_common_), Log-Likelihood-Ratio tests (LRT) comparing models of observed rates with evolutionarily constrained models where all traits evolve at a common rate, and p values are presented. Significant pairwise differences are indicated in bold. Inequalities (greater than > or less than <) and equivalent (≈) symbols indicate the direction of differences in the observed evolution rates among traits.

|  | **Pairwise comparison** | **LogL_obs (common)_** | **LRT** | **P** |
| --- | --- | --- | --- | --- |
| **(a) Drosophila** | |  |  |  |
|  | Fertile L_total_ > Non-fertile L_total_ | 7.67 (4.39) | 6.55_(df=1)_ | **0.01** |
|  | Fertile L_total_ ≈ Seminal receptacle | 6.54 (6.47) | 0.15_(df=1)_ | 0.70 |
|  | Fertile L_total_ > Wing length | 25.95 (6.84) | 38.21_(df=1)_ | **<0.001** |
|  | Non-fertile L_total_ < Seminal receptacle | 5.37 (2.17) | 6.38_(df=1)_ | **0.01** |
|  | Non-fertile L_total_ > Wing length | 33.13 (24.31) | 17.65_(df=1)_ | **<0.001** |
|  | Seminal receptacle > Wing length | 23.80 (5.26) | 37.08_(df=1)_ | **<0.001** |
| **(b) Lepidoptera** | |  |  |  |
|  | Fertile L_total_ > Non-fertile L_total_ | 14.22 (10.70) | 7.04_(df=1)_ | **<0.01** |
|  | Fertile L_total_ ≈ Forewing length | 7.31 (6.91) | 0.80_(df=1)_ | 0.37 |
|  | Non-fertile L_total_ ≈ Forewing length | 17.01 (15.48) | 3.06_(df=1)_ | 0.08 |

**ESM Table S4.** Comparisons of evolutionary rates assuming trait covariance in the observed rate matrices of fertile and non-fertile sperm length in Lepidoptera. Evolutionary rates were compared for fertile (eupyrene) and non-fertile (apyrene) sperm total length (L_total_). The sample size (n), observed (σ^2^_obs_) and common (σ^2^_common_) rate matrices are shown. The evolutionary rates in the diagonal of the rate matrix is presented in bold as these illustrate difference or similarities in the observed evolutionary rates among traits and how they differ from the common evolutionary rate (presented in brackets). The Log Likelihood values for the observed (LogL_obs_) and common models (LogL_common_), Log-Likelihood-Ratio tests (LRT), with associated degrees of freedom (df), comparing models of observed rates with evolutionarily constrained models where all traits evolve at a common rate, p values and AIC values for the observed (AIC_obs_) and common (AIC_common_) models are presented. Significant p-values are presented in bold text.

| **Trait** | **n** | **σ^2^_obs_ ( σ^2^_common_)** | | **LogL_obs_**  **( LogL_common_)** | **LRT** _(df)_ | **P** | AIC_obs_  ( AIC_common_) |
| --- | --- | --- | --- | --- | --- | --- | --- |
|  | | Fertile L_total_ | Non-fertile L_total_ |  |  |  |  |
| Fertile L_total_ | 135 | **0.46 (0.31)** | - | 109.01 (87.50) | 43.04_(df=1)_ | **<0.001** | -210.03 (-168.99) |
| Non-fertile L_total_ |  | 0.12 (0.12) | **0.16 (0.31)** |  |  |  |  |

**Figure S1.** Bayesian consensus tree for 19 species of *Drosophila* from the *obscura* group using the mitochondrial gene cytochrome c oxidase subunit II (COII). The scale bar represents the number of substitutions per site along the branches.


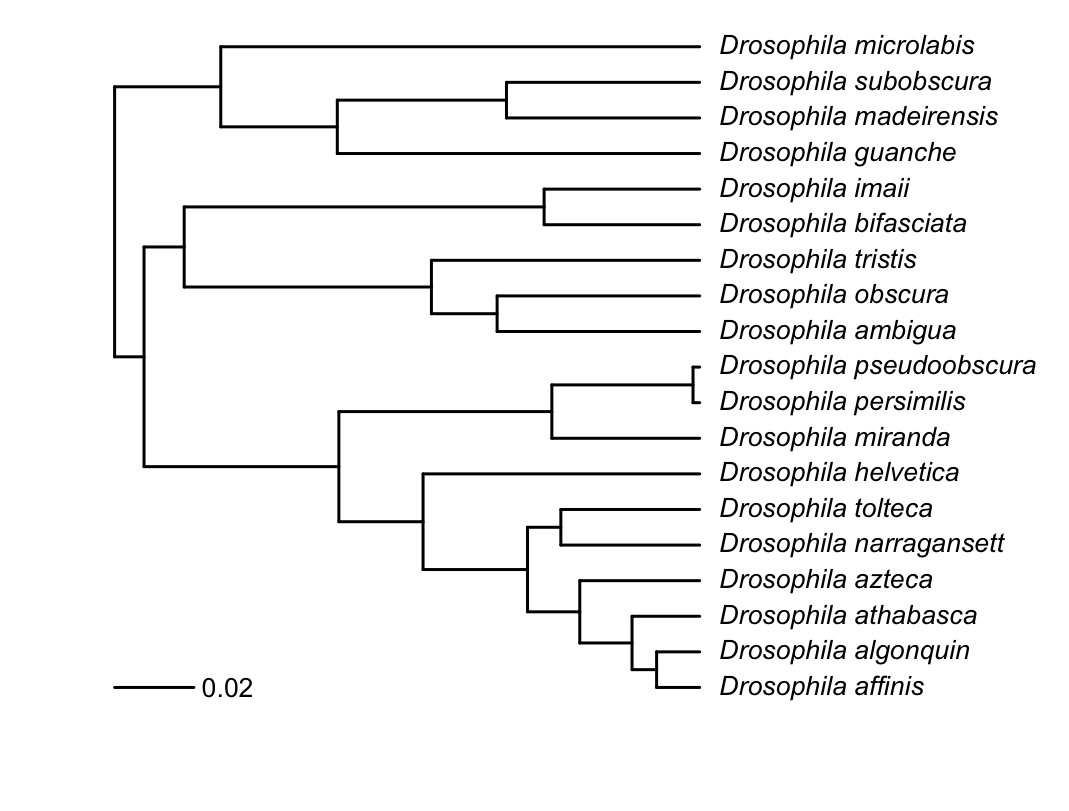


**Figure S2.** Phylogeny of the 12 species pruned from Kawahara et al.’s (5) genomic and transcriptomic-derived Lepidopteran phylogeny where data on fertile and non-fertile sperm lengths were available. The scale bar represents the number of substitutions per site along the branches.


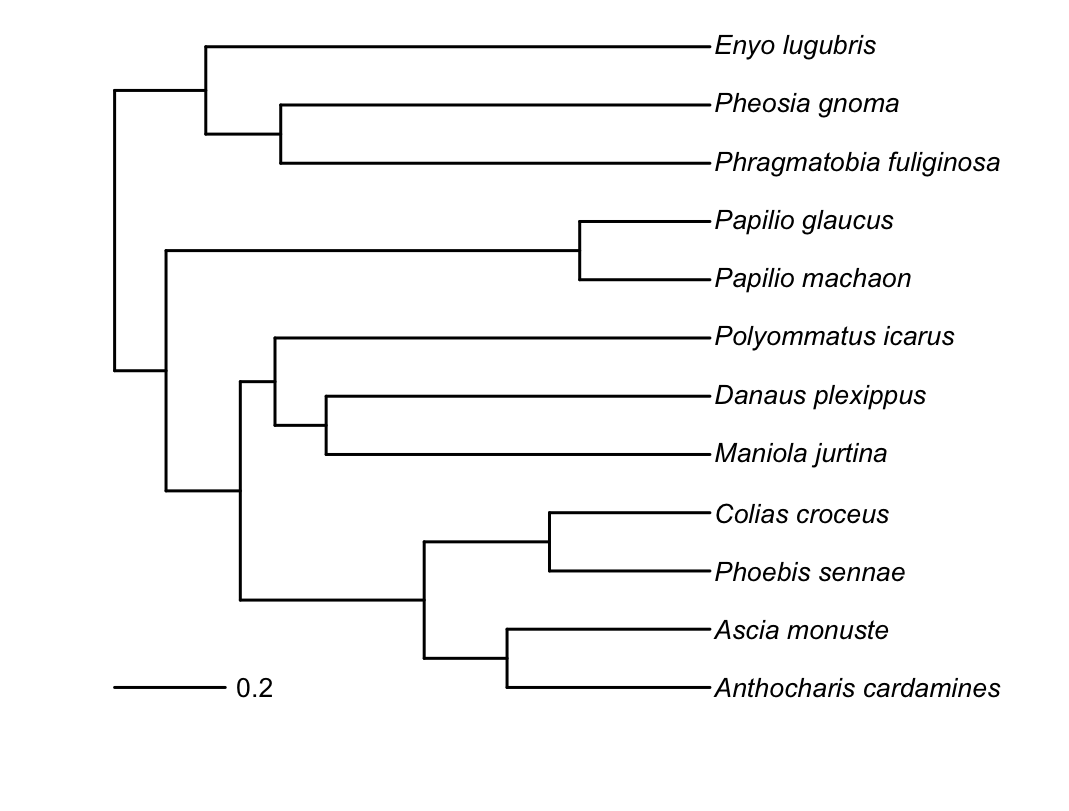

Supplement: ESM Table S1 [file rspb20201286supp1.docx]
